# The Amide Local Anesthetic Lidocaine in Cancer Surgery—Potential Antimetastatic Effects and Preservation of Immune Cell Function? A Narrative Review

**DOI:** 10.3389/fmed.2017.00235

**Published:** 2017-12-20

**Authors:** Thiên-Nga Chamaraux-Tran, Tobias Piegeler

**Affiliations:** ^1^Département d’Anesthésie et Réanimation Chirurgicale, Hôpital Hautepierre, CHU Strasbourg, Strasbourg, France; ^2^Institut de Génétique et de Biologie Moléculaire et Cellulaire, Unité Mixte de Recherche 7104, Centre National de la Recherche Scientifique, U964 Institut National de Santé et de Recherche Médicale, Université de Strasbourg, Illkirch, France; ^3^Department of Anesthesiology and Intensive Care Medicine, University Hospital Leipzig, Leipzig, Germany

**Keywords:** lidocaine, natural killer T-cells, breast cancer, perioperative period, inflammation, immune system

## Abstract

Surgical removal of the primary tumor in solid cancer is an essential component of the treatment. However, the perioperative period can paradoxically lead to an increased risk of cancer recurrence. A bimodal dynamics for early-stage breast cancer recurrence suggests a tumor dormancy-based model with a mastectomy-driven acceleration of the metastatic process and a crucial role of the immunosuppressive state during the perioperative period. Recent evidence suggests that anesthesia could also influence the progress of the disease. Local anesthetics (LAs) have long been used for their properties to block nociceptive input. They also exert anti-inflammatory capacities by modulating the liberation or signal propagation of inflammatory mediators. Interestingly, LAs can reduce viability and proliferation of many cancer cells *in vitro* as well. Additionally, retrospective clinical trials have suggested that regional anesthesia for cancer surgery (either with or without general anesthesia) might reduce the risk of recurrence. Lidocaine, a LA, which can be administered intravenously, is widely used in clinical practice for multimodal analgesia. It is associated with a morphine-sparing effect, reduced pain scores, and in major surgery probably also with a reduced incidence of postoperative ileus and length of hospital stay. Systemic delivery might therefore be efficient to target residual disease or reach cells able to form micrometastasis. Moreover, an *in vitro* study has shown that lidocaine could enhance the activity of natural killer (NK) cells. Due to their ability to recognize and kill tumor cells without the requirement of prior antigen exposure, NKs are the main actor of the innate immune system. However, several perioperative factors can reduce NK activity, such as stress, pain, opioids, or general anesthetics. Intravenous lidocaine as part of the perioperative anesthesia regimen would be of major interest for clinicians, as it might bear the potential to reduce the risk of cancer recurrence or progression patients undergoing cancer surgery. As a well-known pharmaceutical agent, lidocaine might therefore be a promising candidate for oncological drug repurposing. We urgently need clinical randomized trials assessing the protective effect of lidocaine on NKs function and against recurrence after cancer surgery to achieve a “proof of concept.”

## Introduction

Surgery is a main part of the treatment of most cancers. In 2015, 80% of the 15.2 million new diagnosis cases of cancer needed surgery (1). However, the perioperative period might be critical: the long-term follow-up of a cohort of 1,173 patients who underwent mastectomy for breast cancer suggested a bimodal recurrence pattern with an early broad peak at about 18 months after surgery and a second one at about 60 months. If the second peak might be the natural outcome of the breast cancer disease, the first one might result from an early escape of cancer cells from dormancy as well as for recurrence driven by surgery (2). Several competing hypotheses have been proposed to explain this phenomenon (3): surgery might lead to pro-tumorigenic inflammatory changes during the perioperative period, such as increased levels of pro-inflammatory cytokines, prostaglandins, or catecholamines, which might also affect the competence of parts of the innate immune system, such as natural killer (NK) cells, which are crucial for the detection disintegration of circulating tumor cells (CTCs) (4). The surgical stimulus might also increase levels of growth factors like vascular endothelial growth factor (VEGF), basic fibroblast growth factor, transforming growth factor beta (TGF-β), heparin-binding epidermal growth factor*-like growth factor*, or *platelet-derived growth factor*, which have all been linked to tumor growth and metastasis (5–7). By inducing this pro-inflammatory environment in combination with its effects on immune surveillance, surgery itself might therefore enhance cancer cell dissemination and escape from immune surveillance and other hallmarks of cancer such as entrapment, invasion, migration, adhesion, or increasing of NETs (3). Added to the fact that primary tumor removal may promote tumor cell dissemination (8, 9), those effects could enhance establishment of new metastatic foci or accelerate growth of micrometastases (5).

As anesthesia is a key element of the perioperative period, it has been hypothesized that it could possibly also have an influence on cancer recurrence after surgery. On the one hand, some anesthetic and analgesic drug receptors are overexpressed in tumor tissues and are associated with metastasis (10). On the other hand, several retrospective studies have suggested an impact of anesthesia on cancer survival (11); notably, regional anesthesia might be associated with a reduced risk of cancer relapse or recurrence in some studies, although there are also studies reporting no effect (12–14). One of the proposed hypotheses to explain these observations may be related to the anti-inflammatory effects of local anesthetics (LAs) affecting proliferation, migration, or invasion of cancer cells as well (15–17). This review aims at summarizing different properties of amide-linked LAs bearing the potential to exert antitumor or antimetastatic effects. Lidocaine might be of particular interest, as this LA can be used intravenously for multimodal analgesia (18). Perioperative intravenous (IV) lidocaine has already been shown to reduce postoperative pain and opioid requirements (19), appears to be safe, and might not only reduce inflammatory markers but also the length of hospitalization, e.g., after colorectal surgery (20). The drug might therefore be an ideal candidate for a clinical trial evaluating the effects of amide-linked LAs on recurrence after cancer surgery.

## Anti-Inflammatory Effects of LAs *In Vitro* and *In Vivo*

Anti-inflammatory effects of LAs are well-known and have been studied extensively (21). However, the influence of LAs on the integrity of the endothelial barrier might be crucial for a possible inhibitory effect on the generation of metastasis during the perioperative period (14). CTCs—released into the circulation from the primary tumor during surgical removal of the latter—are able to form new (microscopic) metastatic lesions (22, 23), which will finally determine the patient’s fate, even after a complete surgical removal of the primary tumor (24, 25). Endothelial barrier function is mostly regulated by Src protein tyrosine kinase (Src) and the activation of the enzyme will lead to a loss in endothelial barrier integrity *via* phosphorylation of its main substrate caveolin-1 at tyrosine 14 and several subsequent signal transduction pathways finally leading to the disruption of tight junctions and an increase in neutrophil adhesion and transmigration (26, 27), which might also be able to ease the extravasation of CTCs from the circulation (28). Intercellular adhesion molecule-1 (ICAM-1) is crucial for the adhesion and transmigration of neutrophils to the endothelium, thus aggravating the inflammatory response (29, 30). Additionally, phosphorylation of ICAM-1 is not only Src-dependent but also leads to an increase in neutrophil binding and transmigration (31). Src is activated by certain inflammatory cytokines, such as tumor necrosis factor alpha (TNFα), which is released at increasing concentrations—possibly due to surgical stress—during the perioperative period (32, 33). Therefore, the endothelial barrier might be impaired and the formation of new metastatic sites might be favored (14, 34). However, there is evidence that the amide LAs, such as lidocaine and ropivacaine, might be able to attenuate the inflammatory response in the endothelium, which might then lead to a preservation of endothelial barrier integrity (35, 36). In a model of experimental acute lung injury triggered by tracheal instillation of bacterial lipopolysaccharide, ropivacaine was able to attenuate the formation of pulmonary edema and neutrophil transmigration, most certainly by decreasing Src and ICAM-1 expression in rats and mice (37, 38). Data from *in vitro* experiments using human lung microvascular endothelial cells incubated with TNFα and ropivacaine or lidocaine suggested that the drugs might be able to preserve endothelial barrier function by inhibiting signal transduction by the cytokine receptor TNFR1, which subsequently also lead to less Src and ICAM-1 activation and/or phosphorylation (39).

Neutrophil transmigration has also been demonstrated to be a factor influencing CTC extravasation and metastasis, as the CTCs might use the activated leukocytes as some sort of “facilitator” for their own transmigration by binding of cancer cell ICAM-1 to neutrophilic CD11b (integrin αM) (40, 41). Therefore—at least in terms of CTC extravasation—it might be beneficial that LAs seem to impair neutrophil activation and priming (42–44).

## Anti-Inflammatory = Antimetastatic?

There is a large overlap between inflammatory signaling pathways found to be crucial in inflammation as well as in cancer (17, 45, 46). For instance, Src kinase is also involved in signal transduction leading to cancer cell migration, cytoskeleton changes, invasion, proliferation, and the extravasation of CTCs (45, 47–49). Src activation and ICAM-1 phosphorylation in cancer cells can not only be induced by incubation with TNFα but also be blocked by clinically relevant concentrations of lidocaine and ropivacaine (16). Furthermore, the inhibition of Src activation by amide LAs also has an impact on the activation of Akt and focal adhesion kinase (15), a pathway which might also have a crucial role in triple negative breast cancer and is currently investigated for the development of new targeted therapies (48). A decrease in TNFα-induced secretion of cancer cell matrix metalloproteinase 9—an enzyme necessary for the degeneration of the extracellular matrix by malignant cells (50)—in combination with a subsequent decrease in invasiveness *in vitro* has also been linked to the inhibition of Src activation by the LAs (15). Interestingly though, depending on the cell type used, the observed effects on cancer cell invasiveness have both been shown to be either independent (Src-dependent mechanism in non-small-cell lung cancer cells) or dependent (sodium channel variant Na_v_1.5 in colon cancer cells) on the blockade of the voltage-gated sodium channel (VGSC) (16, 51). Cancer growth might also be affected by LAs, as lidocaine has been shown to induce apoptosis and suppress tumor growth in human breast tumor cells (52) as well as in other tumor cells *in vitro* (15, 16, 53–57). Interestingly, it might also be able to sensitize breast cancer cells against chemotherapeutic drugs (58).

Besides these already well-established *in vitro* effects, a very recent study showed encouraging results regarding a possible inhibition of tumor growth by lidocaine in a xenograft model *in vivo* (54): cells originating from a human hepatocellular carcinoma cell line have been injected subcutaneously into immunocompromised mice, which then have been subject to treatment with lidocaine (30 mg/kg) twice a week injected into the peritoneal cavity. Compared to control, lidocaine treatment was able to reduce the growth of the tumor. Furthermore, it was also found to be as effective as treatment with cisplatin (3 mg/kg, once per week) and was even able to increase the sensitivity of the tumor against cisplatin, which the authors related to an increased induction of apoptosis by the LA (54). In another study, the phenomenon of enhancing the effectiveness of a chemotherapeutic agent has been linked to the fact that lidocaine was demonstrated to induce demethylation of deoxyribonucleic acid in breast cancer cells, thus interfering with the cells’ epigenetics, i.e., their regulation of gene expression (59).

In humans, many studies retrospectively analyzed the effects of the use of regional anesthesia and LAs in patients undergoing cancer surgery. Several studies showed a potential beneficial effect (12, 60–62), while others did not (63–66). So far, no data from adequately powered randomized controlled trials (RCTs) are available and therefore the authors of a recent Cochrane review concluded that there is currently only “inadequate” evidence for a potential beneficial effect of the perioperative use of regional anesthesia in cancer patients (67). However, these inconclusive findings might, at least in part, be explainable by the large heterogeneity of studies, tumors, and patients alike (14). Moreover, the intermediate outcomes of RCT so far have been encouraging: in a pilot study utilizing blood samples from patients undergoing breast cancer surgery (NCT 00418457), the serum of women anesthetized with propofol and paravertebral block induced more apoptosis in a triple-negative breast cancer cell line compared to serum derived from women exposed to sevoflurane and an opioid-based regimen (68). In addition, these two different anesthetic techniques might also have an impact on serum concentrations of factors contributing to tumor progression after surgery: propofol plus paravertebral block increased the level of TGF-β, whereas sevoflurane together with opioids increased the level of VEGF-C 24 h after surgery (69). But as both TGF-β and VEGF-C are involved in the regulation of tumorigenesis (70, 71), these findings are insufficient to favor any of those two techniques.

## Effects on the Immune System: Implication for Malignant Diseases

### The Central Role of the Immune System in Cancer

Immunosurveillance by the innate immune system plays a crucial role in the early stages of carcinogenesis and is a promising target to treat breast cancer (72, 73). NK cells drive this process and play a key role in detecting abnormal growth and subsequent activation and recruitment of other immune cells to eliminate cancer cells (74). Moreover, NK dysfunction can lead to breast cancer progression (75), and restoring NK activity is currently under investigation as part of the treatment regimen for breast cancer (76). A derangement of immune processes is a common event during the perioperative period and might lead to severe disturbances, e.g., of NK cell function with subsequent enhanced dissemination of CTCs (77), as NK cell activity can be impaired for up to 7 days after breast cancer surgery (78). Furthermore, surgery shifts the balance of T-helper (Th)1/Th2 toward the Th2 humoral response, a phenomenon which also exerts pro-tumor actions (79, 80).

### Impact of LAs on Cell-Mediated Immunity

The impact of LAs on antitumor immunity and NK cell function is still contradictory. Thus, a meta-analysis comparing the effect of spinal or epidural anesthesia with general anesthesia failed to demonstrate any enhancement of NK cell function after neuraxial anesthesia, due to a significant degree of heterogeneity of the five eligible studies (81). However, the choice of the anesthetic technique might still have an impact on NK cell function: according to a pilot study from the already mentioned RCT evaluating patients undergoing breast cancer surgery with two different anesthetic regimens (propofol + paravertebral block vs. sevoflurane + opioids only, NCT 00418457), serum of women anesthetized with propofol plus paravertebral block for breast cancer surgery impaired the antitumor activity of NK cells much less than that of women exposed to sevoflurane and opioids (68). Turning more specifically to lidocaine, this LA might also affect NK cell activity differently, depending on the concentration used: from the early 1980s until the 2000s, *in vitro* studies have shown that lidocaine might compromise NK cell activity, but the experimental concentrations tested were high (from 0.2 to 5 mg/ml whereas a level of 5 µg/ml is toxic) and were not compatible with concentrations found after a systemic use of the drug (82–84). However, more recently Ramirez et al. have shown that lidocaine at lower and clinically relevant concentrations (10^−8^ to 10^−6^ M) may instead preserve cytotoxicity of isolated human NK cells (85). Whether LAs might also affect other immune cells, e.g., lymphocytes, remains controversial: proliferation of Jurkat cells, an immortalized human T lymphocyte cell line commonly used to study T cell signaling or the expression of various chemokines, was decreased by lidocaine *via* the induction of apoptosis (86, 87) or *via* a dose-dependent inhibition of the cytokines IL-2 and TNF-α (88). It is difficult to translate those *in vitro* results to a clinical immunosuppressive effect of lidocaine because Jurkat cells are derived from the peripheral blood of a patient with T cell leukemia and express the uncontrolled characteristics of cancer cells (89). More physiologically, lidocaine also had an immunosuppressive effect on isolated mouse T cells derived from Peyer’s patches (90) and reduced the secretion of pro-inflammatory cytokines in freshly isolated peripheral blood T cells (88). In addition, lidocaine inhibited the differentiation of Th1 cell responses of mice dendritic cells (91). However, all these experiments again used excessive lidocaine concentrations and therefore a translation into daily clinical use might be rather difficult. On the contrary, under clinical conditions, Yardeni et al. have shown that intraoperative IV lidocaine in combination with patient-controlled epidural analgesia was able to preserve lymphocyte response to phytohemagglutinin-M compared with a control group receiving only normal saline. This suggests that lidocaine might be able to reduce immune dysfunction as induced by the surgical stimulus (92). These results were later confirmed by Wang et al. who have shown that IV lidocaine might also preserve the balance of Th1/Th2 after radical hysterectomy for cervical cancer, whereas Th1/Th2 imbalance might favor tumor cells to escape immune surveillance and clearance (93). All those clinical data suggested an enhanced effect of lidocaine on immunity and might support its clinical use during the perioperative period. While intraoperative IV lidocaine failed to confirm an opioid sparing effect after breast cancer surgery in two studies (94, 95), those same trials have shown a decrease of the incidence and severity of persistent postsurgical pain at the same time (95, 96). One of the hypotheses argued by the authors to explain those results is the impact of the anti-inflammatory properties of lidocaine.

Because the *in vitro* results remain unclear and the clinical mechanisms of action of lidocaine on immune functions are unsettled, it seems urgent to design a clinical trial to study the impact of IV lidocaine on immune function and cancer surveillance and follow the patient to see if an immune modulation during surgery may have an impact on outcome after breast cancer surgery.

## “From Bench-to-Bedside” Application for IV Lidocaine in Oncology?

We have summarized above a lot of promising data—in particular detailed evidence of plausible direct and putative mechanisms of action—to support a new use of lidocaine in oncologic patients as it bears the potential to serve as a “repurposing candidate” drug (97).

First of all it is a well-known drug, commonly used in multimodal analgesia (98) with a well-established an evaluated toxicologic and pharmacokinetic profile for this purpose (99–101).

However, a number of steps have to be undertaken before repurposing IV lidocaine for oncologic diseases. Unfortunately, there is no reliable clinical evidence of its oncological effects available right now. Therefore, we urgently need randomized controlled clinical trials to test the hypothesis of lidocaine’s antitumor effects at clinically relevant doses as suggested by the large amount of *in vitro* and *in vivo* evidence.

So far, the oncological properties of lidocaine were mainly assessed by retrospective studies or secondary analyses of patients enrolled in published clinical trials which were not powered and designed to study other effects or outcome parameters (14). In order to test the hypothesis that lidocaine might have an antitumor or antimetastatic effect, patients would have to be randomized to receive IV lidocaine at relevant doses for perioperative analgesia or placebo. Possible outcome measures could, for instance, be the disease-free survival after a long-term follow-up or the impact of lidocaine on inflammatory or NK cytotoxic functions after surgery. Identifying subgroups of procedures and surgeries where patients are responsive to lidocaine would be useful to demonstrate a protective effect of this LA against recurrence and metastasis. This strategy might help to avoid the inconsistent results of studies on protective effect of regional anesthesia (14). Thus, patients undergoing colorectal or breast cancer surgery could be of interest as those surgeries imply a high risk of local or distant relapse, even after achieving a complete surgical resection (102). Additionally, some phenotypes of those cancers overexpress one of the main targets of lidocaine: the VGSC which might also be involved in the process of metastasis (103).

## Conclusion

Due to its large therapeutic margin, strong anti-inflammatory properties and potential beneficial impact on the innate immune surveillance system, lidocaine might be an ideal candidate for drug repurposing in cancer, which might potentially affect the patients’ outcome dramatically. Besides the already proven favorable effects of perioperative IV lidocaine application on, e.g., post-operative pain and inflammation, patients with (breast) cancer might also benefit from an antimetastatic effect, ideally associated with a subsequent increase in recurrence-free and overall survival. However, due to the fact that there is currently not enough clinical evidence to support this hypothesis, we urgently call for clinical trials evaluating the effects of perioperative lidocaine during cancer surgical period to answer the question once and for all: is there a beneficial effect or not?

## Author Contributions

Both authors contributed equally to this work.

## Conflict of Interest Statement

The authors declare that the research was conducted in the absence of any commercial or financial relationships that could be construed as a potential conflict of interest.
